# The experiences of nurse educators in implementing evidence-based practice in teaching and learning

**DOI:** 10.4102/hsag.v23i0.1177

**Published:** 2018-11-29

**Authors:** Gloria N. Mthiyane, Debbie S. Habedi

**Affiliations:** 1 Department of Health Studies, University of South Africa, South Africa

## Abstract

**Background:**

Nurse educators have a vital role to mentor student nurses in relation to developing evidence-based practice (EBP) skills, accessing research products and participating in research projects. This requires more innovative teaching approaches that promote active participation, creativity and critical thinking in students such as online teaching and learning, accessing electronic resources, video conferencing and research-based teaching and learning.

**Aim:**

To determine the nurse educators’ experiences in implementing EBP in teaching and learning, and to describe the importance and benefits of EBP teaching and learning to the nursing profession, especially for nurse educators and student nurses.

**Setting:**

Two chosen campuses from Umgungundlovu Health District under the KwaZulu-Natal College of Nursing (KZNCN) and offering a 4-year R425 training programme.

**Methods:**

Qualitative research design and methods were followed in conducting the study. A non-probability purposive sampling technique was used to access the sample of 12 nurse educators. Data were collected using semi-structured interviews, the interview guide, and the digital voice recorder.

**Results:**

Data were analysed manually, following a content thematic approach and two themes emerged as challenges experienced by nurse educators with the implementation of EBP in teaching and learning and benefits and value of EBP in teaching and learning. Findings revealed that, although most of the nurse educators are supportive and displayed a positive attitude towards implementing EBP in teaching and learning, the level of knowledge and skills was questionable. This was coupled with a lack of motivation and commitment towards research.

**Conclusions:**

Evidence-based practice has an essential potential role to play through incorporating more practice-based evidence of nurse educators in teaching and learning implementation. The nurse educators should use EBP to ensure that student nurses receive high-quality nursing education.

## Introduction

Evidenced-based practice (EBP) is described by Melnyk et al. (2012:410) as a problem-solving approach to clinical decision-making in health care. Evidence-based practice integrates the best evidence from well-designed studies with the clinicians’ expertise, including internal evidence from patient assessments and practice data, and patients’ preferences and values. Melnyk et al. (2012:410) further revealed that implementation of EBP leads to a higher quality of care, improved patient outcomes, and decreased health care costs.

Evidence-based practice teaching and learning has become an important function for nursing education. Research is used as an instrument in developing new teaching and learning strategies. Nurse educators are guided by evidence-based practice in teaching and on research reports. Evidence-based practice in learning is also based on research, meaning that students learn by using research findings. Research-based teaching and learning encourages and stimulates critical thinking for students (Felicilda-Reynaldo & Utley 2015:91).

Although EBP is known to improve health care quality, decreasing costs and empowering nurses, the challenge is the way in which it ensures successful implementation by the nurses (Levin et al. 2011:21). The implementation of successful EBP education serves the function of developing practitioners who value EBP and have the knowledge and skills to implement such practice (Lehane et al. 2017:8).

## Background

Nurse educators play a key role in creation of opportunities for implementing EBP and in facilitating the implementation process. The question now is how to foster implementation of EBP. The most important factor that is likely to help nurses and nurse educators to adopt EBP is the provision of adequate training in EBP (Heikkila et al. 2017:3). The importance of embedding EBP in nurse education programmes cannot be underestimated if EBP and its positive patient outcomes are to be realised in health care settings. According to Felicilda-Reynaldo and Utley (2015:93), one nurse educator noted that ‘nurse graduates will be prepared to facilitate a transformation of the health care system culture by implementing practice review and revision consistent with evidence-based nursing (EBN) research’. The above-mentioned statement highlights the importance of incorporating EBP throughout the curriculum to prepare students for future success in using EBP in their professional nursing practice (Felicilda-Reynaldo & Utley 2015:93). Mackey and Bassendowski (2016:54) indicated that utilising nursing best practice guidelines, reviewing and implementing applicable research evidence, and taking advantage of technological advances are ways in which nursing can move forward as a well-informed discipline.

Malik, McKenna and Griffiths (2015a:158) in Australia reported that integrating EBP into undergraduate nursing education and preparing future nurses to embrace EBP into clinical practice becomes crucial in today’s complex and evolving health care environment. The study further implies that the role that EBP plays in the practical lives of student nurses will depend on the degree to which it is promoted by academics; the extent to which it is incorporated in course objectives, content and assessments; and its application within the clinical setting (Malik et al. 2015a:158). In this way, nurses’ willingness to carry out research projects, as well as to utilise the research findings effectively in practice is enhanced.

Traditional teaching methods and learning styles still dominate in nursing education and these strategies do not encourage critical thinking among the nursing students; therefore, these methods will not prepare student nurses to make sound clinical judgements in practice (Subhan 2014:35). With the nursing profession experiencing many changes on contemporary issues, nursing students need to be prepared for these challenges. Using EBP as one of the teaching and learning strategies will qualify student nurses to become excellent critical thinkers and solve problems in the clinical area. However, EBP remains a relatively new concept to nursing, and there is limited literature available addressing the incorporation of EBP into nursing curricula, especially at the undergraduate level (Malik et al. 2015a:158). In South Africa, there is a dearth of literature in the implementation of EBP in nursing education.

## Purpose

To determine the experiences of nurse educators in implementing EBP in teaching and learning, and to describe the importance and benefits of EBP in teaching and learning in the nursing profession, especially for nurse educators and student nurses.

### Questions

- What are the experiences of nurse educators regarding implementation of EBP in teaching and learning?
- What recommendations may be made for nurse educators to implement EBP in teaching and learning?

## Design and methods

### Design

A qualitative, explorative, descriptive research design, using non-probability purposive sampling, focusing on understanding the social settings, facilitating the exploration of relationships and human experience within the research setting was followed. It also enabled face-to-face, personal contact in data collection (Moule & Goodman 2014:175). Participants selected and interviewed were individuals who were more knowledgeable about the phenomenon and qualified enough to answer the question at hand.

### Population

The total target population was 62 nurse educators in June 2017.The sample comprised 12 nurse educators in possession of a nursing education qualification, registered by South African Nursing Council (SANC) as nurse educators and employed by the KwaZulu-Natal Department of Health. The focus of the study was on nurse educators involved in classroom teaching at the two chosen campuses from Umgungundlovu Health District under the KwaZulu-Natal College of Nursing (KZNCN) and offering a 4-year R425 training programme.

### Sampling method

The sampling process followed the non-probability method using a purposive sampling technique to select the participants. This type of sampling assisted the researcher to select those homogeneous participants who knew most about the phenomenon under study (Brink, Van der Walt & Van Rensburg 2012:139). All nurse educators who met the inclusion criteria were requested to volunteer for participation in the study. Clinical nurse educators were excluded from the study. The sample size was 12 participants and was based on collecting detailed data to address the research questions, objectives, and the purpose of the study.

### Data collection

Data were collected in June 2017 using semi-structured interviews, interview guide and the digital voice recorder. To ensure anonymity, code numbers were allocated to each participant. The researcher kept all collected and private information shared safely locked and/or encrypted in the researchers’ computer for 5 years. Participants were informed of the presence of the research assistant during the interview process and all participants granted the researcher permission to record interviews.

Semi-structured individual interviews were conducted using the self-developed interview guide and digital voice recorder as data-collection instruments. The interview guide was developed in English based on the research purpose, research objectives, research questions and literature review. It comprised predetermined open-ended questions to get in-depth information about participants’ experiences and was pre-tested before conducting the main study to determine the feasibility of the instrument. All interviews were captured on the digital voice recorder with the help of the research assistant who had training in doing qualitative interviews prior to data collection.

### Data analysis

Data were analysed manually following a content thematic analysis approach which involved a thorough review of all recorded information that the researcher obtained during data collection (Brink et al. 2012:194). The researcher listened to recorded interviews repeatedly. All recorded data were transcribed into written format. Transcriptions were read thoroughly and repeatedly; common themes and categories of information were identified and grouped together. Electronic files for each theme were created and labelled, allowing for ease of access and management of data. Discussion and interpretation of the findings commenced thereafter.

### Trustworthiness

Strategies employed to ensure the quality of data included the following measures of trustworthiness as suggested by Lincoln and Guba’s framework (Polit & Beck 2017:560).

*Credibility*: Data were collected through face-to-face interviews, directly from the participants using a digital voice recorder and they were given a chance to confirm data before the final written report. Participants were provided with the opportunity to review the researchers’ interpretation of data.

*Transferability*: Sufficient description and interpretation of data was supported by the relevant literature so that it can be easily transferrable and applicable to other settings.

To ensure *dependability*, the researcher ensured that data collected remained stable over a period of time; meaning that information related to data collected would remain unchanged over time. Therefore, data were collected using the digital voice recorder with a memory card and all other information were properly stored and kept safely.

The use of actual quotations from the participants in the discussion of the report supports the *confirmability* of data.

### Ethical considerations

Ethics approval certificate (reference number HSHDC/537/2016) was obtained from the Research Ethics Committee of the Department of Health Studies, University of South Africa (UNISA). Permission to conduct the study was granted by the KwaZulu-Natal Department of Health, KwaZulu-Natal College of Nursing, and both principals of the colleges. Participants were informed about the study and that their participation was voluntary and they were advised of their rights to withdraw at any time. All participants signed informed consent forms, their responses were kept confidential and anonymous and their identities were not divulged or disclosed during reporting of the findings.

### Biographical data of the participants

Participants were from three race groups: African, Indian and Coloured.^1^ They were all women. Their years of experience ranged between 5 and 30 years, occupying the ranks of junior nurse educator, senior nurse educator and Head of Department. The highest qualifications were a Bachelor’s, Honour’s or Master’s degree in Health and Nursing Science Studies as indicated in Table 1.

**TABLE 1:** Demographic information of the sample.

Criterion	Frequency	Percentage
Position
Junior	5	41.6
Senior	5	41.6
Head of department	2	16.6
Work experience
0–5 years	1	8.3
5–10 years	4	33.3
Above 10 years	7	58.3
Highest qualification
Bachelor’s degree	4	33.0
Honour’s degree	2	1.0
Master’s degree	6	50.0
Doctoral degree	0	0.0
Professorship	0	0.0

## Themes, categories and findings

The following themes and categories emerged during data analysis and are summarised in Table 2.

**TABLE 2:** Themes and categories.

Themes	Categories
Theme 1: Challenges with implementation of EBP teaching and learning	1.1. Time constraints 1.2. Lack of and poor access to relevant resources 1.3. Current teaching approaches 1.4. Lack of knowledge and skills by nurse educators 1.5. Student characteristics
	Theme 2: Benefits and/or values of EBP in teaching and learning	2.1 Keeping up to date with current information 2.2 Preparing student nurses to engage in EBP 2.3 Improving quality care for patients 2.4 Reducing health care delivery costs

EBP, evidence-based practice.

### Theme 1: Challenges with implementation of evidence-based practice in teaching and learning

The findings revealed that nurse educators are experiencing certain challenges with the implementation of EBP in teaching and learning. They include time constraints, lack of and poor access to relevant resources, the use of traditional teaching approaches which are still dominant, nurse educators’ lack of or poor knowledge and skills, and the quality of nursing students. Emmanuel et al. (2011:22) in their study on developing EBP among student nurses in the United Kingdom identified similar challenges that prevented the nurses from successfully using EBP, which included poor access to facilities and information, lack of experience and little confidence in using computers.

Schoonees, Rohwer and Young (2017:11) reported that the challenges experienced by lecturers at a sub-Saharan African academic institution were a lack of time by the programme to dedicate to evidence-based health care, lack of evidence related to a specific field, lack of student motivation and the students’ schooling background.

#### Category 1.1: Time constraints

This study indicated that, for the nurse educators to successfully implement EBP in teaching and learning, more time is required in the library to search for information, for reading the literature, and for preparation and presentation of the content. This applied to both the nurse educators and student nurses. This statement is supported in the study conducted by Emmanuel et al. (2011:22), which indicated that the use of EBP by the professionals requires accessing and integrating a number of different resources which could be time consuming. Lack of time was also mentioned as a major barrier when trying to access and review any of the evidence (Emmanuel et al. 2011:22).

Nurse educators indicated that time is not allocated or distributed fairly between library, theory and practice. The prescribed time in the curriculum does not provide sufficient time for students to visit the library to search for information. The location of the library also contributed to time limits, neither campus under study having a library on site. On one campus, the library is 8 km away from the campus. At the other campus, the library is within the hospital premises and not on the campus. These are both medical libraries, which means that they are accessed by all hospital staff. The following statements were reported by the participants with regard to time allocation, prescribed time, and preparation time:

‘Yes, yes, the students have library, the problem that we encounter when they are in block is that they do not have enough time to go to the library because the libraries are not situated in the facilities, like colleges they must go out and find information that is our challenge for now.’ (Participant 1, Junior, 40 years old)

‘So, you find that even the time that is allocated for library the moment they walk out to go to the library, you find that library is in use full of doctors then that is a challenge and you find that now when they come back they are somehow late for classes so that is a challenge.’ (Participant 2, Junior, 42 years old)

‘Maybe we can move from what we have already, because you know generally there is allocated time for clinical exposure, allocated time for theory. Maybe we can look at both these components and also allocate time for self-directed learning so that it comes in as part of the curriculum, like a prescribed kind of thing, like for example saying that the students must have so many assignments, must have so much library time.’ (Participant 3, Senior, 50 years old)

A study conducted by Malik et al. (2015b:50) on nurse educators and clinical nurse coaches and specialists regarding their perceived knowledge, skills, attitudes and contextual factors affecting EBP, revealed that insufficient time prevented appraisal of literature on a regular basis and the searching of research reports. There is no time to find and read research articles. Again, in circumstances where shortages of staff exist, allowing staff adequate time to complete the requisite reading to update their clinical or EBP knowledge or to attend continuing nursing education is not always possible (Boswell & Cannon 2017:20).

#### Category 1.2: Lack of and poor access to relevant resources

It is clear that for the nurse educators to implement EBP in teaching and learning, special resources must be available and accessible to both nurse educators and student nurses for successful implementation. Evidence is available in various forms, such as books, videos, journals, and articles. Access to evidence comes through the libraries, computer laboratories with Internet access and mobile technology.

Participants responded this way when asked about their resources:

‘For instance, if you look at the in-house, what you need to make your teaching learning process smarter, is sometimes not available.’ (Participant 11, Senior, 48 years old)

‘It could be available but you cannot access it easily.’ (Participant 8, Junior, 53 years old)

‘No not easily especially with the IT, we are limited for resources; I would have to go to the library to use it.’ (Participant 10, Senior, 59 years old)

‘Eh I don’t think especially with our college, I do not think that we do not, I mean we have enough resources, like for instance we need to have enough resources, the most recent textbooks you know, even the library should make sure that it is well equipped with most recent textbooks, we have the computers so that they have access to the internet which is the problem that we are experiencing that we don’t have with our campus.’ (Participant 4, HOD, 56 years old)

Hussein and Hussein (2014:870) agree with the statements above in their study conducted in Egypt. Nursing education must be committed to the principles of EBP and critical thinking. Such education must provide resources and create a supportive environment for the implementation of EBP in teaching and learning. The fundamentals of teaching student nurses must be based on the best available evidence to recognise and deliver high-quality patient care (Emmanuel et al. 2011:21).

Insufficient financial resources as well as journals, reports and computers for making EBP a reality in theoretical and clinical teaching could affect negatively the nurse educators’ ability to access evidence from various sources. It has been discovered that when nurses are provided with the necessary tools such as smart phones and computers inter alia, they are much more likely to access relevant information and best practice guidelines (Mackey & Bassendowski 2016:53).

#### Category 1.3: Current teaching approaches

The study revealed that, currently, traditional teaching approaches are still dominant in nursing education. The participants identified the following current teaching strategies used for teaching and learning: facilitation, group discussion, simulation, assignments, role play, case study, clinical debates, lecture, self-activities, demonstrations, peer group teaching, problem-solving, group presentations, games, case-based research, videos, reflective journals, portfolio of evidence, and learning package. Most of these approaches lead to technical skills mastery but they do not stimulate the development of critical-thinking skills, as one participant stated that:

‘They promote psychomotor skills; the students get technical skills to do the procedures and more.’ (Participant 9, HOD, 59 years old)

Felicilda-Reynaldo and Utley (2015:94) recommended a change in educational technology for nurse academics, which include the teaching approaches that facilitate critical thinking and provide opportunities for practising evidence-based patient care. The study by Leufer and Clearly-Holdforth (2015:6) mentions that the continuous assessment element of the module comprising an EBP project and the lectures must provide ongoing support to student nurses throughout the research module as they develop their project. Tailored teaching and assessment methods must be implemented within the academy to foster a culture of EBP at undergraduate level and beyond (Leufer & Clearly-Holdforth 2015:7). Teaching of student nurses must be based on the best available evidence to recognise and deliver high-quality patient care (Emmanuel et al. 2011:21).

#### Category 1.4: Lack of knowledge and skills by nurse educators

Other challenges experienced by nurse educators on EBP implementation in teaching and learning were expressed by some of the participants as lack of confidence, lack of motivation, and lack of relevant knowledge and skills about EBP teaching. The following quotes were stated:

‘I think educators need to know how to use EBP research, I think that research for educators is something very important. I am not really completely involved with research, I think they got to understand research, they got to practice research and then, we can bring it to the students.’ (Participant 8, Junior, 53 years old)

‘Maybe the in-service or the education of the staff themselves, the information that they have regarding what problem-based practice or research is all about. They need to be in serviced as well.’ (Participant 10, Senior, 59 years old)

‘The first challenges will be the lack of motivation if they have not been motivated that’s a challenge, because there need to be like more workshops on evidence-based and the importance of using evidence-based, which is the challenge at the present moment because it is not everybody that is well tuned with evidence-based that is the challenge.’ (Participant 4, HOD, 56 years old)

‘Research is very good, it is good and as much as this off the topic you know we hate research, nurses hate research but I think they should be encouraged so we have more EBP and you know encourage the students.’ (Participant 6, Senior, 56 years old)

Although various studies indicate that nurse educators have a positive attitude towards EBP, it has been reported that a positive attitude is increased with advanced educational level, higher academic ranking, years of experience, and a teaching and research role (Hussein & Hussein 2013:609). Therefore, findings suggest that continuing education for nurse educators on the EBP process is necessary to enhance their knowledge and skills in acquiring appropriate research and analytic skills relevant to their teaching specialties (Hussein & Hussein 2013:617).

In an Australian study on integration of EBP into the curriculum, Malik et al. (2015a:158) mention that there is lack of clarity on EBP content and process. Clarity is frequently blurred with research processes and outcomes, which often results in continuation of traditional nursing research courses in the hope of preparing EBP practitioners. Leufer and Clearly-Holdforth (2015:11) state that if optimal patient outcomes are to be maximised, practitioners’ skills and knowledge base must be fostered and enhanced through ongoing education, training and support.

#### Category 1.5: Student characteristics

Nurse educators had a challenge with students’ attitude and perceptions towards EBP, and their lack of computer skills. According to participants, students appear to have preconceived ideas about research in general. These perceptions are negative and overwhelming for students. A negative attitude develops among the student nurses, because students do not understand the importance of EBP. Nurse educators felt that students’ lack of confidence, motivation, and commitment to perform and use research contributed to misperception and their resultant negative attitude. The nursing programme R425 is perceived as being full, overwhelming, with competing priorities of research, clinical, and theoretical workloads.

‘Students feel that it is an added responsibility.’ (Participant 7, Junior, 49 years old)

‘Students that we are teaching, they look at this research as a monster.’ (Participant 12, Senior, 60 years old)

‘So, the experience that I am coming across when I teach is that these students they do not like it.’ (Participant 12, Senior, 60 years old)

‘These students, they think that no eh eh we are overloading them.’ (Participant 5, Junior, 50 years old)

‘Yah there are challenges because at times the learners are lazy, they do not want to do work.’ (Participant 11, Senior, 48 years old)

Students’ attitudes and perceptions can influence EBP learning either positively or negatively. Should students’ roles be clearly defined early during training, so that they understand the importance of EBP, a positive attitude develops within them. According to Malik et al. (2015a:158), the role that is played by EBP in the practical lives of nursing students depends on the degree to which it is promoted by academics, the extent to which it is incorporated into course objectives, content and assessments; and its application to clinical practice.

In a study by Hickman, Kelly and Phillips (2014:603) on exploring ways to optimise the uptake of EBP to undergraduate nurses, findings indicated that students were initially hesitant and reluctant towards the subject of research. However, as they advanced with the subject their perceptions and beliefs changed to understanding the relevance of the research subject and its importance in improving patient outcomes.

Students’ lack of computer skills is another challenge that nurse educators encountered. Students are fully dependent on them for these skills and more time is needed to orientate students on how to use the computers. The use of EBP requires searching of information from the Internet. To accomplish this one must be able to use computers to search for information. These are the quotes from participants:

‘When you ask students for instance, initially we had challenges with them not knowing, because you find that at school they never did anything that has to do with computers, like any classes and now when they come here they have to sit in front of the computer, they do not even know what the mouse is, you know.’ (Participant 3, Senior, 50 years old)

‘So, I would love to see in the initial stage of training, this newly employed student nurses to have a basic course in computer literacy so that when we send them to fish information or explore they have the skills on how to like do internet surfing, go through the specific web, SANC web and etc., so that we are offload with them relying on us with computer skills.’ (Participant 9, HOD, 59 years old)

Increasing competence in information literacy is the foundation for EBP; this provides nurses with the skills to be literate consumers of information in an electronic environment (Emanuel et al. 2011:22). According to Emmanuel et al. (2011:23), advances in IT have had a radical impact on health care delivery and nurse education. The use of sophisticated equipment and electronic assessment care packages requires nurses to be competent in IT skills.

For students to be ready for EBP learning, they need to have good basic computer skills and to develop the right attitude towards learning using EBP. Their learning styles must support the inculcation of EBP. Fostering a culture of EBP in student nurses is essential to delivering effective health care (Leufer & Clearly-Holdforth 2007:4) and it is also important that student nurses be equipped with EBP knowledge and skills to encourage evidence-informed decision-making after graduation (Schoonees et al. 2017:1). Students should have the right attitude for functioning as independent health care providers (Young et al. 2015:354). Nursing education institutions (NEIs) have a responsibility to provide support to student nurses in the development of relevant IT skills. This calls for services of the librarian to provide skills to student nurses in searching electronic databases for evidence. Clinical librarians in health services have to promote information literacy in the workplace. Health-related degree programmes need to adopt and incorporate EBP methods into their curricula to prepare practitioners in training for their future professional roles and responsibilities (Spring & McCuskey 2010:249).

The recommendations that were made by the participants concerning student nurses were about creating good networking between the clinical practitioners and nurse educators to support the student nurses, providing students with basic computer skills so that they can work independently, and introducing a reward system as motivation for student nurses who are doing well.

### Theme 2: Benefits and value of evidence-based practice in teaching and learning

Although some studies indicated that the main barrier to EBP is lack of value for EBP (Melnyk et al. 2012:415), this study revealed that participants also believed that integration of EBP in teaching and learning as the best way to follow. It showed ways in which EBP teaching and learning could benefit the nurse educator, the nursing education discipline, student nurses, patients, and the employer or institution. Evidence-based practice is associated with critical-thinking skills which must be developed among the student nurses, and lifelong learning for nurses, to expand their knowledge and quality of care related to patient and health care facilities (Felicilda-Reynaldo & Utley 2015:91-92). The following categories of information offer the benefits that come with implementation of EBP in teaching and learning.

#### Category 2.1: Keeping up to date with current information

Participants believed that EBP empowers and enriches the nurse educators and student nurses with up-to-date information, thus keeping their knowledge current. They also believe that EBP is based on researched international studies, and grounded on expert knowledge. These remarks were made by participants:

‘I think it is the best way to do your teaching and learning because you have evidence to base your information on.’ (Participant 1, Junior, 40 years old)

‘The other thing, you will find that you do not have to waste more time trying to give information to the students because everything is there.’ (Participant 1, Junior, 40 years old)

‘Again, that are not going to be having people that would be having something that is outdated, again when we actually engage in that we also update ourselves as lecturers.’ (Participant 12, Senior, 60 years old)

Nurse educators displayed a positive attitude and appeared to be very supportive of EBP teaching and learning. Reinforcing this statement, Mehrdad et al. (2012:507) reported that nurses’ knowledge and attitude towards EBP contribute to its implementation in health care. Therefore, nurse educators and nursing students need to keep up to date with current evidence for use in practice. Current practices relied on by senior nurses and nurse educators are frequently based on personal experience, tradition, intuition, and organisational policies or protocols, rather than on evidence (Malik et al. 2015b:47).

There is a belief that the use of up-to-date scientific findings will improve the quality of care for patients and fill the gap between research, theory, and practice (Mehrdad et al. 2012:509). It is important that nurses use evidence to ensure their practice is up to date and based upon the best available research (Hickman et al. 2014:604). Malik et al. (2015a:159) emphasise that the health professionals are required to be using up-to-date knowledge and evidence to guide their decisions, hence improving patient care. In order for nursing education to keep up with the changes in nursing and health care, such professionals should be immersed in EBP (Felicilda-Reynaldo & Utley 2015:92).

#### Category 2.2: Preparing student nurses to engage in evidence-based practice

According to the nurse educators, student nurses lack interest in research. However, once they become involved with research activities, the tendency is for students gradually to develop more interest and a concomitantly positive attitude towards research. Equally, increased motivation and eagerness to learn is noticed. The following statement was reported:

‘But when they come back from the library with those articles you could see that they are now developing interest you know, they are developing interest, they seem to understand it is like now there is that curtain that has fallen away so they can see now the light in this research.’ (Participant 6, Senior, 56 years old)

Hickman et al. (2014:603) agree that developing the EBP capabilities of the emerging nursing workforce is essential in improving patient outcomes, promoting organisational efficiencies and creating a satisfying work environment. Engaging students in applying evidence into professional practice is a critical role of nursing faculties. This results in student nurses gaining confidence in the use of research and EBP to inform decision-making. Hickman et al. (2014:603-604) further reveal that students demonstrated the ability to identify, criticise, and translate research to practice owing to the increased understanding. Students also understood the difference between research and EBP and how one informs the other. Therefore, teaching concepts of EBP to student nurses to enable them to recognise and deliver high-quality care that is evidence-based is a key outcome of all nurse education programmes (Leufer & Clearly-Holdforth 2015:4).

#### Category 2.3: Improvement to quality care for patients

Evidence-based practice allows for individualised care, improvement in quality care leading to quick recovery of patients and shortened period of hospitalisation which saves money for patients. Participants stated the following:

‘Research helps in the improvement in the quality of care and in how quick the patient recovers or how long he stays ill in the ward.’ (Participant 3, Senior, 50 years old)

‘I think the benefits, the benefits are, because EBP is more patient centered I think and therefore it is beneficial and also sometimes it becomes meaningful, you can even individualize the care to the patient based on that EBP and the outcome of that so I think it is beneficial in that way but eh those are the benefits I think and also improvement, improvement in patient care.’ (Participant 8, Junior, 53 years old)

When patient care is informed by sound evidence, it results in better and more affordable care towards people and the community. Nursing evolved from a series of dictated tasks to a holistic care approach which requires evidence that is developed into guidelines. These guidelines support nurses to promote individualised care (Emmanuel et al. 2011:22).

Evidence-based practice is a patient-centred approach as it influences patient care and minimises the theory-practice gap with nursing (Mackey & Bassendowski 2016:53). The main aim of EBP is to optimise outcomes for patients and clients by selecting interventions that have the greatest chance of success (Leufer & Clearly-Holdforth 2015:4).

#### Category 2.4: Reducing health care delivery cost

The use of EBP leads to improvement in the quality of care, thus reducing patient stay in hospital. Although quality is sometimes expensive, it comes with positive implications for health care facilities, for example, when patients receive quality care, their recovery is quicker and the numbers of days spent in hospital are reduced, resulting in reduction of patient-care costs. Therefore, the employer and institutions benefit positively from the use of EBP because of the end result, which is improvement in the patient outcomes. One participant reported that:

‘You know I feel like with EBP teaching and learning, it will be more important to use more in the clinical area.’ (Participant 10, Senior, 59 years old)

For the health institutions to provide effective services, their functioning, planning, policies, and guidelines are informed by researched evidence. The following statements were reported:

‘I think EBP will be an excellent way to have proficient and efficient nurses in the ward.’ (Participant 5, Junior, 50 years old)

‘You get in improvement, quality improvement in nursing care and you find that with research, research helps in the improvement in the quality of care and in how quick the patient recovers or how long he stays ill in the ward.’ (Participant 4, HOD, 56 years old)

Reducing health care costs requires that nursing and health care services be based on best current available evidence. Improved cost-effectiveness of the health institutions allows the administrators the ability to negotiate for better sponsorship and incentives from health care funders and insurers (Levin et al. 2011:22).

## Discussion

Evidence-based practice teaching and learning is not explicitly implemented. The current curriculum (R425) does not have any specific guidelines for EBP teaching and learning; it only requires that research processes and steps to conduct research projects be taught to student nurses. For KZNCN, the research module is taught at third-year level, and it is covered under the Community Nursing Science component. The learner study guide only requires the nursing students to conduct and present a mini research project. There is no mention of EBP skills or competencies that must be acquired or achieved.

The researchers concluded that the information generated as experiences of nurse educators is not directly linked to EBP in teaching and learning: it was more about teaching and learning of research in general. Nevertheless, EBP depends for its success on researched information. Research is conducted to develop evidence that may be used for EBP. The utilisation of researched evidence in practice is known as EBP. It is never easy to separate research from EBP because one complements the other. However, it remains the responsibility of the NEIs to encourage student nurses to promote and deliver EBP from the onset of training; therefore, the principles of EBP should be introduced to students as part of pre-registration education (Emmanuel et al. 2011:21).

Lack of resources was one of the challenges mentioned by most of the nurse educators, mainly the physical resources like lack of or poor access to computers and libraries. Non-availability of Internet access, current books, journals, and articles also contributed to poor access to relevant evidence needed for successful implementation of EBP teaching and learning.

From the list of teaching strategies identified by nurse educators, it was clear that traditional teaching strategies are still dominant in nursing education. Such strategies do not promote or stimulate critical-thinking skills and EBP competencies to student nurses. A teaching method is understood to be a particular technique a teacher uses to help learners gain the knowledge which they need and to achieve a desired outcome. A desired outcome in this context is the development of critical thinking, problem-solving and good decision-making skills for student nurses, which can be achieved by the use of EBP in teaching and learning as one of the innovative teaching strategies.

Though it transpired that most nurse educators were supportive and had a positive attitude towards the implementation of EBP in teaching and learning, their level of knowledge and skills was questionable; there was uncertainty in understanding the use of EBP in teaching and learning; and a lack of motivation and commitment towards research was evident. Malik et al. (2015b:46), in a tertiary health care facility in Victoria, Australia, investigated the perceptions of nurse educators on factors promoting EBP and perceived barriers to facilitating EBP in a clinical setting before developing an educational programme. The findings revealed that nurse educators had positive attitudes towards EBP implementation. However, they demonstrated a lack of knowledge and skills in appraising and incorporating evidence into practice. Nurse educators cannot be expected to teach what is not known to them, therefore; they must be equipped with knowledge and skills so that they can teach EBP to produce professional nurses who are competent in evidence-based care (Melnyk et al. 2012:416).

## Limitations

It may not be possible to generalise the findings to all nurse educators because the study was limited to nurse educators who work in the public institutions under Umgungundlovu Health District, and who only provide R425 comprehensive 4-year programmes at two campuses. Therefore, findings cannot be generalised to private institutions like universities, other districts, provinces or even nationally and for other nursing programmes.

## Recommendations

The current curriculum (R174) should be reviewed and restructured to allow for early introduction of EBP principles at the onset of training. In addition, online and telematics studies together with the use of mobile technologies should be included in the curriculum.

Relevant adequate resources should be made available and accessible to nurse educators and nursing students. Access to sufficient clinical facilities that are appropriate for the achievement of the outcomes of the programme should also be considered.

Nurse educators should be supported through in-service training, workshops and affiliation to journal clubs to improve their knowledge and skills regarding EBP competencies.

## Conclusion

The findings revealed both the challenges and benefits that come with the use of EBP in teaching and learning skills for the nursing education discipline, thus paving the way for the implementation of the suggested recommendations. Evidence-based practice has an essential potential role to play through incorporating more practice-based evidence of nurse educators in teaching and learning implementation. Therefore, nurse educators should use EBP to ensure that student nurses receive high-quality nursing education.

## Footnotes

1 The author understands and acknowledges the sensitivity of the terms used and prefers to use as classified according to the Republic of South Africa’s Government Application Form for Employment in order to avoid misinterpretation of the study results.
